# Trends in Coronary and Structural Heart Interventions in Switzerland over the Last 16 Years and Impact of COVID-19: Insights from the National Swiss PCI Survey

**DOI:** 10.3390/jcm11247459

**Published:** 2022-12-15

**Authors:** Max Wagener, Jasper Boeddinghaus, Oliver Gaemperli, Lorenz Räber, Fabian Nietlispach, Pascal Meier, Olivier Muller, Daniel Weilenmann, Raban Jeger

**Affiliations:** 1 Department of Cardiology, Cardiovascular Research Institute Basel (CRIB), University Hospital Basel, 4031 Basel, Switzerland; 2 Department of Cardiology, University Hospital Galway, H91 YR71 Galway, Ireland; 3 BHF Centre for Cardiovascular Science, University of Edinburgh, Edinburgh EH16 4TJ, UK; 4 Department of Cardiology, Heart Clinic Hirslanden, 8032 Zurich, Switzerland; 5 Department of Cardiology, Bern University Hospital, 3010 Bern, Switzerland; 6 Heart Centre Im Park, Hirslanden Clinic Im Park, 8027 Zurich, Switzerland; 7 Cantonal Hospital Freiburg, 1752 Villars-sur-Glâne, Switzerland; 8 Department of Cardiology, University Hospital Vaud, 1011 Lausanne, Switzerland; 9 Cantonal Hospital St. Gallen, 9007 St. Gallen, Switzerland; 10 Department of Cardiology, Triemli Hospital Zurich, 8063 Zurich, Switzerland; 11 Faculty of Medicine, University of Basel, 4001 Basel, Switzerland

**Keywords:** percutaneous coronary intervention, structural intervention, COVID-19, Switzerland

## Abstract

Background: Considering the global burden of cardiovascular disease, we analysed trends in interventional coronary and structural procedures over the past 16 years (2005–2021), using continuous data from the Swiss national registry. Methods: Based on a standardised questionnaire, data on coronary and structural interventions in Switzerland were assessed by the Working Group Interventional Cardiology of the Swiss Society of Cardiology (SSC). Here, we analysed the trend of annually performed interventions from 2005 to 2021 in Switzerland and the impact of the COVID-19 pandemic. Results: We observed a constant increase in the total number of cases (including coronary angiographies (CA) and percutaneous coronary interventions (PCI)) from 36,436 cases in 2005 to 56,555 cases in 2021 (+55%). With 18 cases in 2007, TAVI procedures have increased to 2004 cases in 2021. During the early phase of the COVID-19 pandemic in 2020, a slight decrease in CAs and PCIs of 9.15% was observed. In contrast, we did not observe an impact of the COVID-19 pandemic on the number of no TAVI procedures. Most importantly, all cause in-hospital mortality for coronary interventions before and during the peak of the COVID-19 pandemic was comparable (1.4% vs. 1.3%). Conclusion: Over a 16-year period, we observed an upward trend in diagnostic and therapeutic procedures for coronary as well as structural heart disease, with only a small short-term impact of the COVID-19 pandemic on interventions and a similar procedure-related in-hospital-mortality in Switzerland.

## 1. Introduction

With 3.8 million deaths per year in Europe, cardiovascular disease is still the leading cause of death in Europe, accounting for 46% and 39% of all deaths in women and men, respectively [1]. With ischemic heart disease (IHD) being responsible for 47% of all cardiovascular deaths, rising numbers in coronary diagnostics and interventions were observed across Europe [1]. In a similar way, the global burden of valvular heart disease, evolving treatment options and an increasing body of evidence led to a worldwide increase in transcatheter aortic valve implantations (TAVI) [2,3]. In this context, we evaluated general trends in interventional cardiology in Switzerland over the past 16 years, using the annual assessment coordinated by the Working Group Interventional Cardiology of the Swiss Society of Cardiology [4,5,6,7,8,9,10,11].

Due to missing or shifts in healthcare resources, the COVID-19 pandemic largely influenced the diagnosis and treatment of cardiovascular disease, with large variations among different healthcare systems [12,13,14,15]. Beside the 16-year trend, we evaluated the impact of the COVID-19 pandemic on interventional coronary and structural procedures in Switzerland [10,11,16,17].

## 2. Methods

### 2.1. Assessment of Coronary and Structural Interventions

A standardised online questionnaire via the Survio© platform (Survio s.r.o., Brno, Czechia) was sent to all interventional centres in Switzerland. Alternatively, the questionnaire was made available as a .pdf file. General information regarding centres with catheter laboratories on-site, indication for intervention, caseload of interventional coronary artery and structural heart disease procedures, as well as periprocedural aspects and in-hospital-mortality (if available) were assessed. A complete list of variables can be found in the Supplementary Materials. Only diagnostic coronary angiographies (CA) are referred to as “CA”. Percutaneous coronary interventions (PCI), which always included the diagnostic angiography as well as the intervention, are referred to as “PCI”.

### 2.2. Statistical Analysis

Data are expressed as numbers and percentages. Official data from the Swiss Federal Statistical Office was used to analyse data in its demographic context. Considering that no personal patient information was assessed, data collection and analysis for in-hospital mortality after interventional procedures were for quality assurance/control purposes only, and no formal approval by local institutional review boards and/or written patient consent was required. All analyses were performed using SPSS for Windows 27.0 (SPSS, Inc., Chicago, IL, USA).

## 3. Results

### 3.1. Demographic Changes & Interventional Centres

In 2005, 208 operators covered 7,459,128 permanent Swiss residents in 27 cardiac interventional centres (five university, nine public non-university and thirteen private hospitals). In accordance to the demographic changes as published by the Federal Statistical Office with 8,736,500 permanent residents in 2021, there are now 39 sites (few of them as a reunited hospital group) covered by 206 operators offering interventional cardiac procedures (three sites did not report their operator numbers) [5,18].

### 3.2. Coronary Interventions

**Figure 1:**
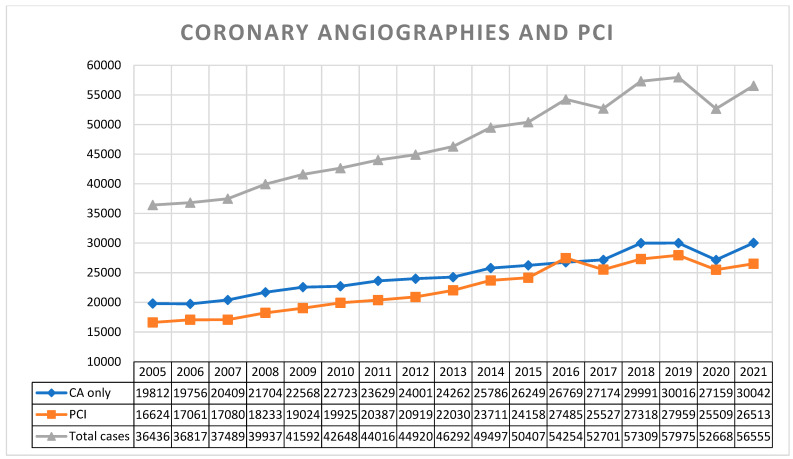
Trends in coronary angiographies and percutaneous coronary interventions in Switzerland from 2005 to 2021. (CA only—coronary angiographies only, without intervention. PCI—percutaneous coronary intervention, including preceding diagnostic angiography).

As illustrated in Figure 1, we observed a constantly rising trend in the total number of cases (CAs + PCIs) during the first decade (2005–2015) of our observation, with 36,436 cases (19,812 CAs and 16,624 PCIs) in 2005 and 50,407 cases (26,249 CAs and 24,158 PCIs) in 2015. After correction for demographic changes, this trend remained true with 488 cases/100,000 residents in 2005 vs. 605 cases/100,000 residents in 2015. For the following years (2016–2021), a peak was reached with 57,975 cases (30,016 CAs and 27,959 PCIs), equalling 674 cases/100,000 residents in 2019.

### 3.3. Impact of COVID-19 on Coronary Interventions

**Figure 2:**
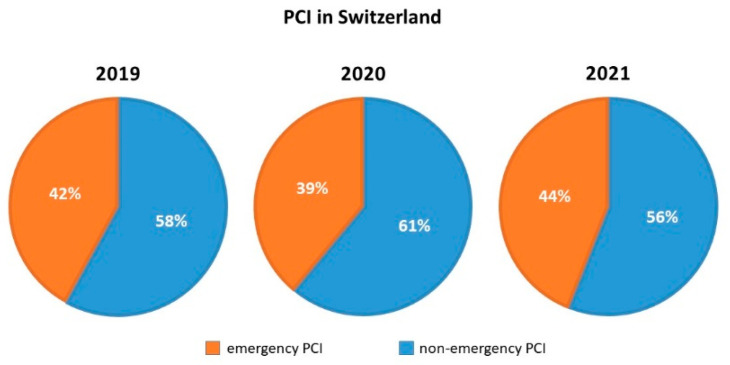
Proportions of emergency and non-emergency PCIs pre (2019), during (2020) and post (2021) the peak of the COVID-19 pandemic. (PCI—percutaneous coronary intervention, including preceding diagnostic angiography).

**Figure 3:**
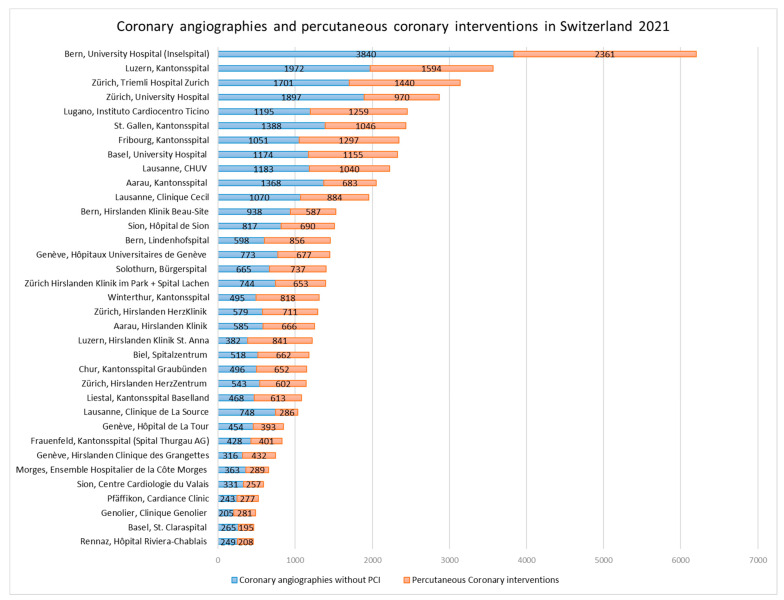
Coronary angiographies and percutaneous coronary interventions in Switzerland 2021, ranked by procedural numbers.

During the COVID-19 pandemic, the federal office of public health mandated a nationwide deferral of non-urgent elective medical procedures, coming into effect on 16 March 2020 [16]. A relief of these restrictions was tolerated from 27 April 2020 on and left under cantonal jurisdiction and control. In comparison to 2019, the total number of cases (CAs + PCIs) dropped by 5307 cases (−9.15%) to an absolute number of 52,668 cases in 2020 (Figure 1). A slight variation in the proportion of emergency PCIs was noted comparing the years pre (2019), during (2020) and post (2021) the peak of the pandemic, representing 42% (of 27,959 PCIs), 39% (of 25,509 PCIs) and 44% (of 26,513 PCIs), respectively (Figure 2). In 2021, we could observe a recovery of the total number of coronary cases 56,555 (including 30,042 CA and 26,513 PCI), representing a minus of 2.45% to the pre-COVID-19 year in 2019 (Figure 1 and Figure 3).

### 3.4. Operator Volume, Access Routes and Indications for Coronary Interventions

**Figure 4:**
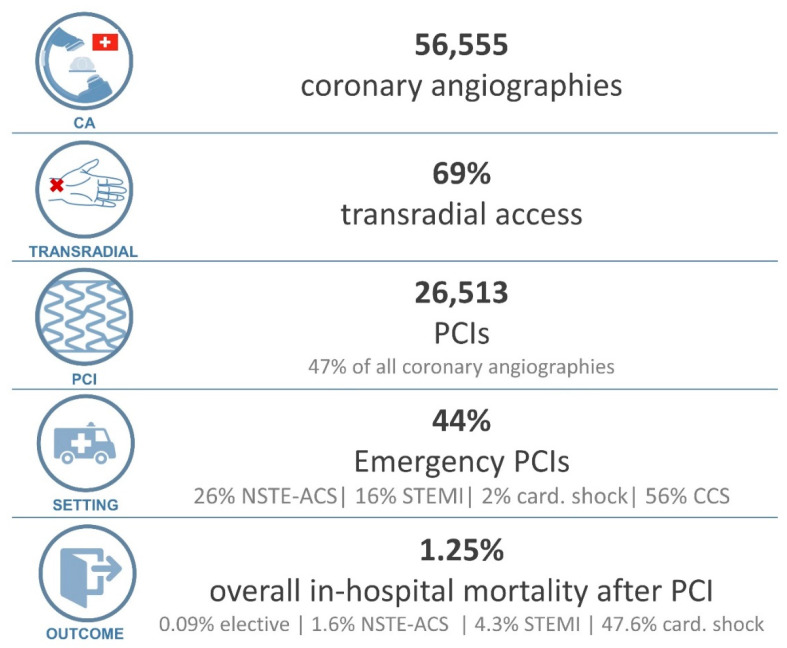
Coronary interventions in 2021: From top to bottom: box 1: Coronary angiographies; box 2: radial access rate; box 3: percutaneous coronary interventions; box 4: Indication for PCI; box 5: outcomes after PCI. (CA: coronary angiographies, card: cardiogenic, CCS: chronic coronary syndrome, PCI: percutaneous coronary intervention, NSTE-ACS: non ST-elevation acute coronary syndrome, STEMI: ST-elevation myocardial infarction).

With a relatively stable number of interventional cardiologists over the years, the caseload per operator increased from 175 total cases/per operator in 2005 (208 operators), to 275 cases per operator in 2021 (206 operators). The evolution of interventional methods and interventional guidelines led to a transition from a mainly femoral access rate (>85% in the early observation period), to a radial access rate of 69% in 2021 (Figure 4). The indications for PCI in 2021 were as follows: 55.7% chronic coronary syndrome (CCS including CTO), 26.0% non-ST-elevation acute coronary syndrome (NSTE-ACS), 16.4% ST-elevation myocardial infraction (STEMI), 1.9% patients with cardiogenic shock, Figure 4 (data on indication for intervention reported from 34 centres).

Details on interventional techniques such as type of stents used, auxiliary revascularisation methods as well as on hemodynamic support used can be found in the Supplementary Tables S1 and S2.

### 3.5. Structural Interventions

**Figure 5:**
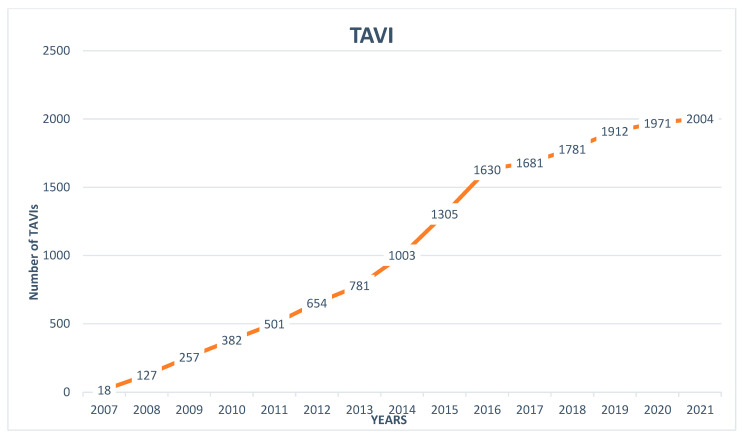
Trends in transcatheter aortic valve implantations in Switzerland from its introduction in 2007 to 2021.

**Figure 6:**
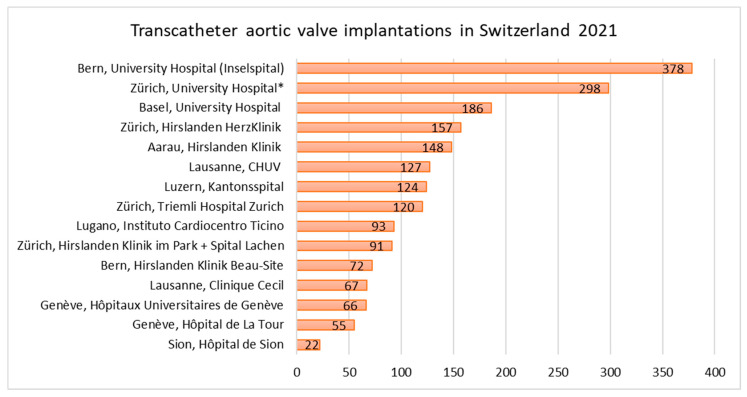
Transcatheter aortic valve implantations in Switzerland, ranked by procedural numbers. * From the 298 TAVIs at the University Hospital Zurich, 109 TAVIs were performed in cooperation with the Cantonal Hospital St. Gallen.

TAVI was introduced in Switzerland in 2007 with 18 cases. Until 2021, a rising trend to a total of 2004 TAVIs in 2021 was observed (Figure 5). The COVID-19 pandemic did not influence the number of TAVI procedures with 1912, 1971 and 2004 procedures in 2019, 2020 and 2021, respectively. In 2021, 93.7% of TAVIs were delivered via femoral access, followed by trans-carotideal (2.3%), trans-apical (1.6%), trans-subclavian (1.4%), direct-aortic (0.9%) and trans-caval (0.1%) access (data missing or incomplete for 3 of 15 centres). The number of TAVIs per individual centre ranged from 22 to 378 procedures per centre (Figure 6). Details on TAVI procedures, other valvular and non-valvular interventions in Switzerland in 2021 can be found in the Supplementary Tables S3–S5.

### 3.6. Outcomes

In the pre-pandemic reference year, 2019, overall in-hospital mortality after PCI was 1.4%. Relevant differences in mortality were noted depending on indication for the PCI (0.1% elective, 1.8% non ST-elevation acute coronary syndrome, 5.4% ST-elevation myocardial infarction and 25.1% in patients with shock at the moment of presentation) [10]. Neither during the peak of the pandemic (2020), nor in the year after the peak of the pandemic (2021), a clinically relevant change in overall mortality was noted. In 2020, overall procedure-related in-hospital mortality after PCI was 1.36% (0.08% elective, 1.6% non ST-elevation acute coronary syndrome, 4.1% ST-elevation myocardial infarction and 42% in patients with shock at the moment of presentation) [11]. In 2021, similar outcomes, with a 1.25% in hospital mortality after PCI were observed (Figure 4). From 2019 to 2021, the PCI related mortalities for CCS, Non-ST-Elevation ACS and STEMI ranged from 0.08–0.1%, 1.6–1.8% and 4.1–5.4%, respectively. Of note, in 2019, 14 centres, and in 2020, 24 centres reported their data on in-hospital mortality.

## 4. Discussion

We report three major findings. First, over the last 16 years, we can observe rising numbers in CA, PCI as well as TAVI in Switzerland. Second, the COVID-19 pandemic had only a minor impact on PCI and no impact on TAVI in Switzerland. Third, in-hospital procedure-related outcomes remained mainly unchanged during the COVID-19 pandemic.

The rising trends in catheter-based coronary and structural interventions are in accordance with the observations from different national registries in Europe. In France, a rise in CA and PCI of +8% and +10% between 2010 to 2015 was observed [19]. In Austria, a country of a comparable population size to Switzerland, similar rising trends in CA and PCI (+6.5% in total cases) were reported from 2012 to 2017 in the ANCALAR registry [20]. In contrast to the Swedish SCAAR Registry with <20% of patients undergoing PCI for chronic coronary syndrome, in Switzerland the main indication for PCI in 2021 was chronic coronary syndrome, with 55.8% [21]. Whether the findings of the ISCHEMIA and the long-term-data from the COURAGE trials will change these trends is yet to be defined [22,23].

Considering the Swiss trend in TAVI numbers, France and Austria experienced similar steep rises in intervention numbers after the introduction of TAVI [19,20]. In France from 2013 to 2015, a rise from 2396 to 5732 TAVIs was observed [19]. Over the period of 5 years, a rise from 432 to 1016 TAVIs in 2017 was observed in Austria (after correction for population size: 11.52/100,000 in comparison to 19.81/100,000 in CH) [20,24].

In contrast to the decline in incidence of disease and mortality from ischemic heart disease in central European countries, similar to France and Austria, we observed a rise in diagnostic and therapeutic invasive coronary procedures [25]. Several factors may explain these rising trends. First, despite the declining incidence trends of ischemic heart disease, the general burden of cardiovascular disease in Europe remains high [1]. Second, the demographic changes in Switzerland with a growing population over the observed period may contribute to the rising interventional numbers [18]. Not only have we observed a rise in absolute, but also in relative numbers, thus a rise in invasive coronary procedures (CA + PCI) from 488 cases per 100,000 permanent Swiss residents in 2005 to 644 cases per 100,000 residents in 2021 was observed [18]. Third, facilitated access to coronary interventions through a broader network of interventional centres, but also the technical development (e.g., the transition from bare metal stents to drug eluting stents) and better safety profiles of modern interventional approaches (e.g., radial access) inducing lower thresholds for referral are contributors to rising interventional numbers [26,27,28]. Considering the impact of the COVID-19 pandemic, invasive diagnostic and therapeutic procedures for coronary heart disease only declined by 9.2% in Switzerland in the year 2020 and numbers recovered in 2021 (only −2.45% compared to 2019). This lays in contrast to the observations from Germany reporting a reduction in catheter laboratory activity of around 35% during the COVID-19 pandemic [12,13]. The number of TAVI procedures seems not to have been affected by the COVID-19 pandemic in Switzerland. Both findings may be explained by a liberal management of the pandemic by the Swiss Federal Office of Public Health (FOPH). Based on hospital capacities, only a short deferral of non-urgent medical procedures and therapies was mandated from the 16 March 2020 to the 26 April 2020 [16]. The influence of different healthcare systems, country size and government policy on the diagnostic and therapy of coronary and structural heart disease may become evident by data from the USA. In the USA, a significant association between a hospital’s COVID-19 disease burden and a reduction in PCI and TAVI volumes was noted for 2020, with a reduction of −55% and −64% for PCI and TAVI, respectively [15].

In the CoVCAD study, covering a population of 6 million people in central Germany, a significant increase in cardiovascular mortality by 8% was observed in parallel to a reduction of catheter laboratory activity by 35% [12]. In combination with an increase of in-hospital mortality in patients presenting with acute coronary syndromes, this leads to the assumption that the deferral and late referral of cardiac patients during the lockdown have caused worse cardiovascular outcomes during the pandemic [12]. In accordance with the German findings, northern Italy, one of the hardest hit regions in Europe by the pandemic, reported significantly lower rates of emergency admissions of patients with acute coronary syndromes at the beginning of the pandemic [29]. Furthermore, an Italian, nationwide, multicentre analysis, showed a 48.4% reduction of admissions for acute myocardial infarction during the first week of COVID-19 pandemic in comparison to the same week in the previous year (*p* < 0.001) [30]. In the mentioned period, case-fatality for STEMI patients (13.7%) was significantly higher than in the pre-pandemic year (RR 3.3, *p* < 0.001) [30]. Lower admission rates, as well as system delays due to the COVID-19 pandemic may explain this phenomenon [30]. In their meta-analysis, Sabatino et al. described a significant interaction of pre-existing cardiovascular risk factors and disease, with the primary endpoint of death (*p* < 0.001), of note a high heterogeneity was noted among the included trials (I^2^ = 99.7 for case fatality rate) [31].

In contrast, we did not observe a relevant change of in-hospital procedure-related mortality of patients undergoing coronary angiography or percutaneous coronary interventions in Switzerland. There was no rise of in-hospital mortality for STEMI patients, with 5.4%, 4.1% and 4.3%, in the years 2019, 2020 and 2021, respectively [10,11]. These findings are well in line with the findings from the Swedish SCAAR-Registry [14]. Although a small decrease in referral was observed, there was no increase in short-term case fatality in this registry [14]. We observed only slight variations in rates of emergency PCI varying from 42% to 39% and back to 44% of all PCI. In Germany, emergency PCI represented 45% during the peak of the pandemic (March and April 2020) vs. 36% of all PCI in the same non-pandemic period in 2019 [12].

In conclusion, over a 16-year period, we observed a rising trend in diagnostic and therapeutic procedures for coronary as well as structural heart disease, with only a small impact of the COVID-19 pandemic on interventions and a similar procedure-related in-hospital-mortality in Switzerland.

## 5. Limitations

This analysis is limited by its voluntary design, and despite scheduled reminders, not all centres provided their data during the time of data collection. Considering the fact that data from 92% of Swiss interventional centres were assessed and mostly small centres did not report data, our data are representative on a national level. Outcome data was not reported from all centres included in our analysis, thus the findings on mortality are of limited generalisability.

## Data Availability

Original data was assessed with the Survio© online database, an SSL encrypted, cloud based software (ISO/IEC 27001:2013). For analysis, data was securely saved on the access restricted server of the Department of Cardiology, University Hospital Basel.

## Supplementary Materials

The following supporting information can be downloaded at: www.mdpi.com/article/10.3390/jcm11247459/s1, Table S1. Details on access and types of stents, scaffolds and/or drug coated balloons used in Switzerland 2021. Table S2. Auxiliary coronary revascularisation techniques, quantification methods of coronary stenosis and hemodynamic support devices used in 2021. Table S3. Transcatheter aortic valve implantations (TAVI) in Switzerland 2021. Table S4. Transcatheter valvular interventions other than TAVI in Switzerland 2021. Table S5. Non-valvular, catheter based cardiac interventions in 2021. File S1. SWISS PCI Survey 2021 (questionnaire).
