# Supplementary material for: Trends in Coronary and Structural Heart Interventions in Switzerland over the Last 16 Years and Impact of COVID-19: Insights from the National Swiss PCI Survey
Source: J Clin Med. 2022 Dec 15;11(24):7459. doi: 10.3390/jcm11247459 (PMC9783484; doi:10.3390/jcm11247459)
Supplement: Supplementary file 1 [file jcm-11-07459-s001.zip › File S1. SWISS PCI Survey 2021 (questionnaire).pdf]

INTERVENTIONAL CARDIOLOGY – SWISS STATISTICS 2021

Dear coordinator

Please fill in the forms with the data of your center by **February 28th 2022**.

If you can't provide numbers for a specific variable (e.g. for outcome data), please type in "**9999**" instead of "0", in order to differentiate not available from no cases/events.

Thank you very much and best Regards,

Raban, Max and Jasper

1 Catheterisation center (name of hospital and city)

2 E-mail address of the coordinator:

3 Number of cath labs

4 Number of PCI operators (number of persons, not full-time equivalent)

5 Centre with cardiac surgery (yes or no)

◯ Yes ◯ No

6 Name of software used for catheterisation database

7 Number of coronary angiographies WITHOUT PCI

8 Number of coronary angiographies WITH PCI

9 Number of PCI for NSTE-ACS (Unstable Angina or NSTEMI)

10 Number of PCI for STEMI

11 Number of PCI for cardiogenic shock (as per clinical diagnosis)

12 Number of PCI for chronic total occlusions (CTO)

13 • Number of CTO with antegrade recanalization (attempted or succeeded)

14 • Number of CTO with retrograde recanalization (attempted or succeeded)

15 Number of radial access for coronary angiography

16 Number of cases with Bare Metal Stents (BMS) - not number of stents!

17 Number of cases with Drug-Eluting Stents (DES) - not number of stents!

18 Number of cases with self-expandable stents (DES or BMS) - not number of stents!

19 Number of cases with bioabsorbable scaffolds (Absorb/Magmaris/etc.) - not number of stents!

20 Number of cases with bifurcation dedicated stents - not number of stents!

21 Number of cases with drug-coated balloon only - not number of balloons!

22 Number of cases with Rotablator

23 Number of cases with shockwave balloon - not number of balloons!

24 Number of cases with thrombus aspiration

25 Number of cases with distal protection device (filters)

26 Number of cases with Fractional Flow Reserve (FFR)

27 Number of cases with instant wave free ratio (iFR)

28 Number of cases with intravascular ultrasound (IVUS)

29 Number of cases with intravascular Optical Coherence Tomography (OCT)

30 Number of cases with intra-aortic balloon counterpulsation (IABP)

31 Number of cases with Impella

32 Number of cases with Extracorporeal Membrane Oxygenation (ECMO)

33 Number of cases with other left ventricular assist devices (e.g. Tandem Heart)

34 Number of cases with aortic valvuloplasty without percutaneous valve replacement

35 Number of cases with Transcatheter Aortic Valve Implantation (TAVI)

36 • Transfemoral TAVI (total number)

37 • Transapical TAVI (total number)

38 • Transsubclavian TAVI (total number)

39 • Direct aortic TAVI (total number)

40 • Transcarotid TAVI (total number)

41 • Transcaval TAVI (total number)

42 Number of cases with use of cerebral embolic protection device during TAVI

43 Number of cases with occlusion of paravalvular leakage

44 Number of cases with percutaneous transvenous mitral valvuloplasty

45 Number of cases with transcatheter mitral edge-to-edge repair devices (e.g. Mitraclip)

46 Number of cases with transcatheter direct mitral annuloplasty (e.g. Cardioband)

47 Number of cases with transcatheter indirect mitral annuloplasty (e.g. Carillon)

48 Number of cases with transcatheter mitral valve replacement (TMVI, e.g. Tendyne)

49 Number of cases with transcatheter tricuspid valve intervention

50 Number of cases with PTA / Stenting pulmonary artery

51 Number of cases with transcatheter pulmonary valvuloplasty

52 Number of cases with TPVI (e.g. Melody)

53 Number of cases with alcohol ablation for septal hypertrophy

54 Number of cases with pericardial drainage (ad hoc or scheduled)

55 Number of cases with catheter based therapy of pulmonary embolism

56 Number of cases with catheter-based renal sympathetic denervation for treatment of hypertension

57 Number of cases with coronary sinus reduction

58 Number of cases with PFO Closure

59 Number of cases with ASD Closure

60 Number of cases with VSD Closure

61 Number of cases with LAA Closure

62 IN-HOSPITAL MORTALITY - Overall after any intervention (coronary and not-coronary, number of cases)

63 IN-HOSPITAL MORTALITY - Overall after any PCI (number of cases)

64 IN-HOSPITAL MORTALITY - after PCI for stable coronary artery disease (number of cases)

65 IN-HOSPITAL MORTALITY - after PCI for NSTE-ACS (Unstable Angina or NSTEMI, number of cases)

66 IN-HOSPITAL MORTALITY - after PCI for STEMI (number of cases)

67 IN-HOSPITAL MORTALITY - after PCI for cardiogenic shock/cardiac arrest (as per clinical diagnosis, number of cases)

68 IN-HOSPITAL MORTALITY - after TAVI (number of cases)
