# Supplementary material for: Trends in Coronary and Structural Heart Interventions in Switzerland over the Last 16 Years and Impact of COVID-19: Insights from the National Swiss PCI Survey
Source: J Clin Med. 2022 Dec 15;11(24):7459. doi: 10.3390/jcm11247459 (PMC9783484; doi:10.3390/jcm11247459)
Supplement: Supplementary file 1 [file jcm-11-07459-s001.zip › jcm-2072452-supplementary.pdf]

Supplementary Materials

Table S1. Details on access and types of stents, scaffolds and/or drug coated balloons used in Switzerland 2021. (BMS—bare metal stents; DCB—drug coated balloon; DES—drug eluting stent, PCI—percutaneous coronary intervention).

				Type of Stent Used per Case					
	**Total Cases**	**PCI**	**Radial Access %**	**BMS**	**DES**	**Self-Expandable (DES or BMS)**	**Bioabsorb. Scaffolds**	**Bifurcation Dedicated Stents**	**DCB**
Aarau, Hirslanden Klinik	1251	666	66	0	634	0	0	0	29
Aarau, Kantonsspital	2051	683	52	0	581	0	0	0	48
Baden, Kantonsspital	-	-	-	-	-	-	-	-	-
Basel, St. Claraspital	460	195	34	0	175	0	0	0	9
Basel, University Hospital	2329	1155	76	0	1096	3	0	0	59
Bern, Hirslanden Klinik Beau-Site	1525	587	67	0	536	0	0	0	8
Bern, Lindenhofspital	1454	856	91	0	800	0	0	12	18
Bern, University Hospital (Inselspital)	6201	2361	64	1	2093	0	0	0	220
Biel, Spitalzentrum	1180	662	82	0	569	0	0	0	13
Chur, Kantonsspital Graubünden	1148	652	68	0	550	0	0	0	9
Frauenfeld, Kantonsspital (Spital Thurgau AG)	829	401	89	0	386	0	0	0	15
Fribourg, Kantonsspital	2348	1297	51	0	1178	0	0	0	97
Genève, Hirslanden Clinique des Grangettes	748	432	89	0	399	0	0	0	26
Genève, Hôpital de La Tour	847	393	95	0	362	0	0	0	13
Genève, Hôpitaux Universitaires de Genève	1450	677	90	0	599	0	4	0	48
Genolier, Clinique Genolier	486	281		0	-	-	-	-	-
Kreuzlingen, Herz-Neuro-Zentrum Bodensee	-	-	-	-	-	-	-	-	-
Lausanne, CHUV	2223	1040	82	0	917	0	0	0	80
Lausanne, Clinique Cecil	1954	884	33	9	792	0	0	0	0
Lausanne, Clinique de La Source	1034	286	57	0	286	0	0	0	87
Liestal, Kantonsspital Baselland	1081	613	97	0	613	0	13	0	44
Lugano, Instituto Cardiocentro Ticino	2454	1259	87	-	-	-	-	-	-
Luzern, Hirslanden Klinik St. Anna	1223	841	12	0	589	0	0	0	90
Luzern, Kantonsspital	3566	1594	69	1	1230	2	0	1	420
Morges, Ens. Hospitalier de la Côte Morges	652	289	81	0	260	0	0	0	10
Pfäffikon, Cardiance Clinic	520	277	36	0	276	0	0	0	1
Rennaz, Hôpital Riviera-Chablais	457	208	81	0	185	0	0	0	15
Sion, Centre Cardiologie du Valais	588	257	100	0	227	0	0	0	8
Sion, Hôpital de Sion	1507	690	85	0	690	0	0	0	0
Solothurn, Bürgerspital	1402	737	57	0	681	0	0	0	37
St. Gallen, Kantonsspital	2434	1046	98	0	-	-	-	-	-
Winterthur, Kantonsspital	1313	818	73	0	774	0	0	0	49
Zürich, Hirslanden HerzKlinik	1290	711	63	4	586	0	0	0	0
Zürich, Hirslanden HerzZentrum	1145	602	46	0	602	0	0	0	0
Zürich Hirslanden Klinik im Park & Spital Lachen	1397	653	-	1	531	0	0	0	0
Zürich, Privatklinik Bethanien	-	-	-	-	-	-	-	-	-
Zürich, Triemli hospital Zurich	3141	1440	82	0	1357	0	0	0	0
Zürich, Universitätsspital	2867	970	50	0	970	0	0	0	0
Total in %			69	0.07	89.96	0.02	0.07	0.05	6.07

Table S2. Auxiliary coronary revascularisation techniques, quantification methods of coronary stenosis and hemodynamic support devices used in 2021. (ECMO—extracorporal membrane oxygenation; FFR—fractional flow reserve; IABP—intraaortic balloon pump; iFR—instantaneous wavefree ration; IVUS—intravascular ultrasound; LVAD—left ventricular assist device; OCT—optical coherence tomography).

	Revascularisation Techniques, Other Than Balloon Angioplasty				Quantification Methods of the Degree of Stenosis				Hemodynamic Support Devices			
	Rotablator	Shockwave Balloon	Thrombus Aspiration	Distal Protection Device	FFR	iFR	IVUS	OCT	IABP	Impella	ECMO	LVAD
Aarau, Hirslanden Klinik	23	0	0	0	0	0	0	0	0	13	0	0
Aarau, Kantonsspital	5	11	54	6	55	84	9	19	0	15	2	0
Baden, Kantonsspital	-	-	-	-	-	-	-	-	-	-	-	-
Basel, St. Claraspital	5	11	9	0	0	37	0	13	2	0	0	0
Basel, Universitätsspital	12	5	64	0	105	0	7	5	8	39	2	0
Bern, Hirslanden Klinik Beau-Site	3	3	10	0	34	0	0	14	1	0	0	0
Bern, Lindenhofspital	8	3	1	0	0	50	56	0	0	0	0	0
Bern, Universitätsspital (Inselspital)	16	-	133	1	257	1	63	407	0	74	5	0
Biel, Spitalzentrum	17	16	5	0	107	20	10	0	0	0	0	0
Chur, Kantonsspital Graubünden	13	9	48	0	-	38	8	23	0	4	0	0
Frauenfeld, Kantonsspital (Spital Thurgau AG)	3	24	-	-	38	116	-	25	-	-	-	-
Fribourg, Kantonsspital	10	36	92	1	230	15	31	37	5	4	4	0
Genève, Hirslanden Clinique des Grangettes	9	6	6	1	98	0	0	28	0	7	1	0
Genève, Hôpital de La Tour	3	4	19	2	40	40	6	17	1	4	0	0
Genève, Hôpitaux Universitaires de Genève	14	25	67	3	0	132	68	108	10	16	8	2
Genolier, Clinique Genolier	5	3	-	-	140	140	0	13	-	-	-	-
Kreuzlingen, Herz-Neuro-Zentrum Bodensee	-	-	-	-	-	-	-	-	-	-	-	-
Lausanne, CHUV	12	29	78	5	307	-	35	72	2	2	9	-
Lausanne, Clinique Cecil	1	-	-	0	146	0	-	-	0	0	0	0
Lausanne, Clinique de La Source	0	0	0	0	79	0	0	0	0	0	0	0
Liestal, Kantonsspital Baselland	26	8	24	0	0	112	18	67	0	9	0	0
Lugano, Instituto Cardiocentro Ticino	22	25	91	3	90	268	38	31	1	21	5	0
Luzern, Hirslanden Klinik St. Anna	6	51	34	0	112	27	14	0	0	12	0	0
Luzern, Kantonsspital	28	43	-	-	258	-	42	484	15	68	7	0
Morges, Ens. Hospitalier de la Côte Morges	1	8	6	1	89	10	0	25	0	1	0	0
Pfäffikon, Cardiance Clinic	0	5	2	0	0	52	1	0	0	0	0	0
Rennaz, Hôpital Riviera-Chablais	-	4	1	-	39	-	-	6	-	-	-	-
Sion, Centre Cardiologie du Valais	0	0	0	0	115	0	0	5	0	0	0	0
Sion, Hôpital de Sion	0	0	13	1	140	0	0	21	5	0	4	0
Solothurn, Bürgerspital	11	17	6	2	113	0	0	0	0	0	0	0
St. Gallen, Kantonsspital	107	74	133	0	235	16	355	78	8	6	4	0
Winterthur, Kantonsspital	3	11	26	-	10	156	32	26	1	7	0	0
Zürich, Hirslanden HerzKlinik	3	-	8	0	6	182	25	2	3	3	0	0
Zürich, Hirslanden HerzZentrum	5	-	10	-	21	53	30	-	0	5	0	-
Zürich Hirslanden Klinik im Park + Spital Lachen	9	0	4	3	31	154	3	0	7	6	3	0

Zürich, Privatklinik Bethanien	-	-	-	-	-	-	-	-	-	-	-	-
Zürich, Triemli Stadtspital Zürich	5	-	-	-	182	-	0	52	11	23	-	0
Zürich, Universitätsspital	12	54	-	-	240	64	256	194	9	41	10	-
**TOTAL**	**397**	**511**	**962**	**29**	**3317**	**1767**	**1107**	**1772**	**89**	**380**	**64**	**2**

Table S3. Transcatheter aortic valve implantations (TAVI) in Switzerland 2021. (Not mentioned in the table: 1 aortic valvuloplasty without percutaneous valve replacement performed by the St. Claraspital.) * From the 298 TAVIs at the University Hospital Zurich, 109 TAVIs were performed in cooperation with the Cantonal Hospital St. Gallen.

	Aortic Valvuloplasty w/o Valve Replacement	TAVI	Access for TAVI						Cerebral Protection Device	Occlusion of Paravalvular Leakage
			Femoral	Apical	Subclavian	Direct Aortic	Transcarotid	Transcaval		
Aarau, Hirslanden Klinik	0	148	145	0	3	0	0	0	0	0
Basel, Universitätsspital	0	186	164	6	16	0	0	0	86	0
Bern, Hirslanden Klinik Beau-Site	0	72	72	0	0	0	0	0	1	0
Bern, Universitätsspital (Inselspital)	2	378	373	2	0	1	1	1	-	8
Genève, Hôpital de La Tour	1	55	54	0	0	0	1	0	7	0
Genève, Hôpitaux Universitaires de Genève	0	66	64	1	1	0	0	0	63	0
Lausanne, CHUV	1	127	100	0	0	2	25	0	21	3
Lausanne, Clinique Cecil	0	67	59	0	0	0	8	0	0	-
Lugano, Instituto Cardiocentro Ticino	2	93	74	6	1	11	1	0	6	0
Luzern, Kantonsspital	11	124	123	0	1	0	0	0	107	0
Sion, Hôpital de Sion	0	22	22	0	0	0	0	0	5	0
Zürich, Hirslanden HerzKlinik	0	157	-	-	-	-	-	-	28	1
Zürich, Hirslanden Klinik im Park + Spital Lachen	-	91	91	0	0	0	0	0	-	-
Zürich, Triemli Stadtspital Zürich	0	120	110	10	0	0	0	0	-	3
Zürich, Universitätsspital *	-	298	-	-	-	-	-	-	-	-

Table S4. Transcatheter valvular interventions other than TAVI in Switzerland 2021. (PTA—percutaneous transluminal angioplasty; TMVI—transcatheter mitral valve implantation; TPVI—transcatheter pulmonary valve implantation; transcath.—transcatheter).

	**Transcath. Mitral Valve Interventions**					**Transcath. Tricuspid Valve Interventions**	**Transcath. Pulmoary Valve Interventions**		
	**Valvuloplasty**	**Edge-to-Edge Repair**	**Direct Annuloplasty (e.g. Cardioband)**	**Indirect Annuloplasty (e.g. Carillon)**	**Valve Replacement (TMVI, e.g. Tendyne)**		**PTA/Stenting Pulmonary Artery**	**Valvuloplasty**	**TPVI (e.g. Melody)**
Aarau, Hirslanden Klinik	1	50	0	0	0	4	0	0	0
Aarau, Kantonsspital	0	14	0	0	0	5	0	0	0
Baden, Kantonsspital	-	-	-	-	-	-	-	-	-
Basel, St. Claraspital	-	-	-	-	-	-	-	-	-
Basel, Universtitätsspital	1	17	0	0	5	5	0	0	0
Bern, Hirslanden Klinik Beau-Site	0	9	0	0	0	0	0	0	0
Bern, Lindenhofspital	0	0	0	0	0	0	0	0	0
Bern, Universitätsspital (Inselspital)	2	59	0	0	9	36	30		2
Biel, Spitalzentrum	0	0	0	0	0	0	0	0	0
Chur, Kantonsspital Graubünden	0	0	0	0	0	0	0	0	0
Frauenfeld, Kantonsspital (Spital Thurgau AG)	-	-	-	-	-	-	-	-	-
Fribourg, Kantonsspital	-	5	-	-	-	-	-	-	-
Genève, Hirslanden Clinique des Grangettes	0	2	0	0	0	0	0	0	0
Genève, Hôpital de La Tour	0	0	0	0	0	0	0	0	0
Genève, Hôpitaux Universitaires de Genève	1	24	0	0	2	0	18	0	0
Genolier, Clinique Genolier	-	-	-	-	-	-	-	-	-
Kreuzlingen, Herz-Neuro-Zentrum Bodensee	-	-	-	-	-	-	-	-	-
Lausanne, CHUV	2	18	-	-	1	-	1	4	2
Lausanne, Clinique Cecil	0	0	0	0	0	0	0	0	0
Lausanne, Clinique de La Source	0	0	0	0	0	0	0	0	0
Liestal, Kantonsspital Baselland	0	0	0	0	0	0	0	0	0
Lugano, Instituto Cardiocentro Ticino	0	25	0	0	0	4	0	0	0
Luzern, Hirslanden Klinik St. Anna	0	0	0	1	0	0	0	0	0
Luzern, Kantonsspital	2	38	0	0	0	6	0	1	0
Morges, Ens. Hospitalier de la Côte Morges	0	0	0	0	0	0	0	0	0
Pfäffikon, Cardiance Clinic	0	0	0	0	0	0	0	0	0
Rennaz, Hôpital Riviera-Chablais	-	-	-	-	-	-	-	-	-
Sion, Centre Cardiologie du Valais	0	0	0	0	0	0	0	0	0
Sion, Hôpital de Sion	0	0	0	0	0	0	0	0	0
Solothurn, Bürgerspital	0	0	0	0	0	0	0	0	0
St. Gallen, Kantonsspital	0	23	0	0	0	0	0	0	0
Winterthur, Kantonsspital	0	0	0	0	0	0	0	0	0
Zürich, Hirslanden HerzKlinik	2	29	0	0	2	3	0	0	0
Zürich, Hirslanden HerzZentrum	-	-	-	-	-	-	-	-	-
Zürich Hirslanden Klinik im Park + Spital Lachen	2	34	-	-	-	7	-	-	-
Zürich, Privatklinik Bethanien	-	-	-	-	-	-	-	-	-
Zürich, Triemli Stadtspital Zürich	-	8	0	0	3	0	0	0	0
Zürich, Universitätsspital	1	14	0	0	0	0	-	-	-
**TOTAL**	**14**	**369**	**0**	**1**	**22**	**70**	**49**	**5**	**4**

Table S5. Non-valvular, catheter based cardiac interventions in 2021. (PFO—persistant foramen ovale; ASD—atrial septal defect; VSD—ventricular septal defect; LAA—left atrial appendage).

	Alcohol Ablation for Septal Hypertrophy	Pericardial Drainage (Ad Hoc or Scheduled)	Pulmonary Embolism Catheter Based Therapy	Renal Sympathetic Denervation	Coronary Sinus Reduction	Closure Devices			
						PFO CLOSURE	ASD Closure	VSD Closure	LAA Closure
Aarau, Hirslanden Klinik	1	0	0	0	0	20	6	0	22
Aarau, Kantonsspital	2	12	0	0	0	48	3	0	6
Baden, Kantonsspital	-	-	-	-	-	-	-	-	-
Basel, St. Claraspital	-	4	-	-	-	-	-	-	-
Basel, Universitätsspital	6	33	18	3	1	45	7	0	20
Bern, Hirslanden Klinik Beau-Site	0	0	0	0	0	14	-	0	0
Bern, Lindenhofspital	0	5	0	0	0	13	1	0	3
Bern, Universitätsspital (Inselspital)	6	-	45	3	-	125	24	1	100
Biel, Spitalzentrum	0	6	0	0	0	8	1	0	0
Chur, Kantonsspital Graubünden	0	-	0	0	0	6	0	0	4
Frauenfeld, Kantonsspital (Spital Thurgau AG)	-	-	-	-	-	15	-	-	-
Fribourg, Kantonsspital	0	8	5	2	0	25	1	0	18
Genève, Hirslanden Clinique des Grangettes	0	0	0	0	0	2	0	0	0
Genève, Hôpital de La Tour	0	11	0	0	0	19	1	0	2
Genève, Hôpitaux Universitaires de Genève	4	30	0	3	2	36	1	0	6
Genolier, Clinique Genolier	-	-	0	10	0	5	0	0	0
Kreuzlingen, Herz-Neuro-Zentrum Bodensee	-	-	-	-	-	-	-	-	-
Lausanne, CHUV	2	20	-	2	-	37	10	1	6
Lausanne, Clinique Cecil	0	-	0	0	0	-	0	0	-
Lausanne, Clinique de La Source	0	0	0	0	0	2	0	0	0
Liestal, Kantonsspital Baselland	1	8	12	0	0	17	2	0	5
Lugano, Instituto Cardiocentro Ticino	0	19	1	6	2	18	1	0	12
Luzern, Hirslanden Klinik St. Anna	0	1	0	0	0	4	0	0	1
Luzern, Kantonsspital	9	-	2	0	14	66	12	0	25
Morges, Ens. Hospitalier de la Côte Morges	0	2	0	0	0	0	0	0	0
Pfäffikon, Cardiance Clinic	0	3	0	0	0	8	0	0	2
Rennaz, Hôpital Riviera-Chablais	-	-	-	-	-	-	-	-	-
Sion, Centre Cardiologie du Valais	0	0	0	0	0	0	0	0	0
Sion, Hôpital de Sion	0	13	0	3	0	12	1	0	1
Solothurn, Bürgerspital	0	2	19	2	1	13	1	0	17
St. Gallen, Kantonsspital	0	20	0	0	0	35	3	0	5
Winterthur, Kantonsspital	0	5	0	0	0	24	1	0	0
Zürich, Hirslanden HerzKlinik	0	4	0	1	0	19	5	0	12
Zürich, Hirslanden HerzZentrum	-	16	-	1	-	12	-	-	16
Zürich Hirslanden Klinik im Park + Spital Lachen	2	6	-	-	-	52	10	2	16
Zürich, Privatklinik Bethanien	-	-	-	-	-	-	-	-	-
Zürich, Triemli Stadtspital Zürich	2	22	0	0	0	63	16	1	26
Zürich, Universitätsspital	-	-	-	-	-	52	-	-	41
**Total**	**35**	**250**	**102**	**36**	**20**	**815**	**107**	**5**	**366**
